# Genome Sequence of *Acinetobacter baumannii* Strain 5021_13, Isolated from Cerebrospinal Fluid

**DOI:** 10.1128/genomeA.01213-15

**Published:** 2015-10-15

**Authors:** Sunil Kumar, Prashant P. Patil, Samriti Midha, Pallab Ray, Prabhu B. Patil, Vikas Gautam

**Affiliations:** a Department of Medical Microbiology, Post Graduate Institute of Medical Education and Research, Chandigarh, India; b Laboratory of Bacterial Genomics and Evolution, CSIR-Institute of Microbial Technology, Chandigarh, India

## Abstract

We report here the 4.1-Mb draft genome sequence of *Acinetobacter baumannii* strain 5021_13, a cerebrospinal fluid isolate from northern India. This genome information will help studies toward understanding the epidemiology and pathogenicity of this important nosocomial pathogen.

## GENOME ANNOUNCEMENT

*Acinetobacter baumannii* has emerged as one of the most successful pathogens in the health care setting globally, causing a variety of infections that include bacteremia, pneumonia, meningitis, urinary tract infection, and wound infection (1, 2). In India, there are many reports on the emergence of multidrug-resistant strains of *A. baumannii* (3, 4). Infection of the central nervous system by multidrug-resistant *A. baumannii* is difficult to treat and results in a high mortality rate (5). In this study, we report the genome sequence of strain 5021_13, isolated from a cerebrospinal fluid specimen from a patient admitted to the neurosurgery ward of the Postgraduate Institute for Medical Education and Research (PGIMER), Chandigarh, a tertiary-care hospital located in the northern part of India.

High-molecular-weight genomic DNA was isolated using the ZR Fungal/Bacterial DNA MiniPrep kit (Zymo Research) and quantified using a Qubit 2.0 fluorometer (Life Technologies). Illumina sequencing libraries were prepared by using the Nextera XT sample preparation kit (Illumina, Inc., San Diego, CA, USA) with dual indexing adapters from Illumina, as per the manufacturer guidelines. Sequencing libraries were normalized and sequenced by using a 2 × 250-bp paired-end configuration on the in-house Illumina MiSeq (Illumina, Inc.) platform. A total of 2,837,504 reads were generated, amounting to 398,925,392 bp, and were *de novo* assembled using CLC Genomics Workbench version 7.5 (CLC bio, Aarhus, Denmark) into 187 contigs, with a total length of 4,120,995 bp and mean coverage of 96.8×. The assembly has an *N*_50_ of 46,149 bp and average contig length of 22,037 bp, with a mean G+C content of 38.8%. The draft genome was annotated using the NCBI Prokaryotic Genome Annotation Pipeline (http://www.ncbi.nlm.nih.gov/genome/annotation_prok/), and a total of 3,932 coding sequences (CDSs), 2 rRNAs, and 59 tRNAs were predicted.

BLAST analysis of the complete 16S rRNA gene sequence extracted from the genome by RNAmmer version 1.2 (6) revealed 99.87% similarity to type strain *A. baumannii* ATCC 19696^T^. Further average nucleotide identity (ANI) was calculated using JSpecies version 1.2.1 (7). The ANI value between this isolate with the genome of type strain *A. baumannii* ATCC 19696^T^. (8) is 97.4%. For ANI, a 95 to 96% cutoff is used for species delineation, showing that this isolate belongs to *A. baumannii*.

This genome will aid in the whole genome-based phylogenetic and comparative analyses of this important opportunistic pathogen isolated from diverse clinical samples from India and the rest of the world.

### Nucleotide sequence accession numbers.

The draft genome sequence of *A. baumannii* 5021_13 is available in DDBJ/EMBL/GenBank under the accession no. LGYV00000000. The version described in this paper is the first version, LGYV01000000.

## References

[1] Peleg AY, Seifert H, Paterson DL 2008 *Acinetobacter baumannii*: emergence of a successful pathogen. Clin Microbiol Rev 21:538–582. doi:10.1128/CMR.00058-07. 18625687 PMC2493088

[2] Maragakis LL, Perl TM 2008 *Acinetobacter baumannii*: epidemiology, antimicrobial resistance, and treatment options. Clin Infect Dis 46:1254–1263. doi:10.1086/529198. 18444865

[3] Azim A, Dwivedi M, Rao PB, Baronia AK, Singh RK, Prasad KN, Poddar B, Mishra A, Gurjar M, Dhole TN 2010 Epidemiology of bacterial colonization at intensive care unit admission with emphasis on extended-spectrum β-lactamase- and metallo-β-lactamase-producing Gram-negative bacteria–an Indian experience. J Med Microbiol 59:955–960. doi:10.1099/jmm.0.018085-0. 20413621

[4] Taneja N, Singh G, Singh M, Sharma M 2011 Emergence of tigecycline & colistin resistant *Acinetobacter baumannii* in patients with complicated urinary tract infections in north India. Indian J Med Res 133:681. 21727671 PMC3136000

[5] Yang M, Hu Z, Hu F 2012 Nosocomial meningitis caused by *Acinetobacter baumannii*: risk factors and their impact on patient outcomes and treatments. Future Microbiol 7:787–793. doi:10.2217/fmb.12.42. 22702531

[6] Lagesen K, Hallin P, Rødland E, Stærfeldt H, Rognes T, Ussery D 2007 RNammer: consistent annotation of rRNA genes in genomic sequences. Nucleic Acids Res 35:3100–3108. 17452365 10.1093/nar/gkm160 PMC1888812

[7] Richter M, Rosselló-Móra R 2009 Shifting the genomic gold standard for the prokaryotic species definition. Proc Nalt Acad Sci 106:19126–19131. doi:10.1073/pnas.0906412106. PMC2776425 19855009

[8] Davenport KW, Daligault HE, Minogue TD, Bruce DC, Chain PSG, Coyne SR, Jaissle JG, Koroleva GI, Ladner JT, Li P-E, Palacios GF, Scholz MB, Teshima H, Johnson SL 2014 Draft genome assembly of *Acinetobacter baumannii* ATCC 19606. Genome Announc 2(4):e00832-14. doi:10.1128/genomeA.00832-14. 25146140 PMC4153487

